# Overview of Inflammatory and Coagulation Markers in Elderly Patients with COVID-19: Retrospective Analysis of Laboratory Results

**DOI:** 10.3390/life15030370

**Published:** 2025-02-26

**Authors:** Corina Popazu, Aurelia Romila, Marius Petrea, Robert Marius Grosu, Alina-Maria Lescai, Adriana Liliana Vlad, Violeta Diana Oprea, Alexia Anastasia Ștefania Baltă

**Affiliations:** 1 Clinical-Medical Department, Faculty of Medicine and Pharmacy, “Dunarea de Jos” University, Str. Domnească 35, 800201 Galaţi, Romania; corinapopazu@yahoo.com (C.P.); adriana.vlad.mg3.4@gmail.com (A.L.V.); alexiabalta@yahoo.ro (A.A.Ș.B.); 2 Medical Department, Faculty of Medicine and Pharmacy, “Dunarea de Jos” University, Str. Domnească 35, 800201 Galaţi, Romania; aurelia.romila@yahoo.com (A.R.); diana.v.oprea@gmail.com (V.D.O.); 3 Pre-Clinical Department, Faculty of Medicine and Pharmacy, “Dunarea de Jos” University, Str. Domnească 35, 800201 Galaţi, Romania; marius.petrea92@yahoo.com (M.P.); grosurobert21@gmail.com (R.M.G.)

**Keywords:** COVID-19, elderly patients, coagulation markers, inflammatory markers, hypercoagulability, coagulopathy, prognosis

## Abstract

**Background:** Elderly patients with COVID-19 often exhibit a complex interplay between hypercoagulability and coagulopathy, key factors in determining the risk of severe complications and mortality. This study aimed to analyze coagulation and inflammatory markers to identify critical predictors of adverse outcomes in this vulnerable population. **Material and Methods:** The retrospective study was conducted on a sample of 1429 elderly patients (≥60 years) diagnosed with COVID-19, hospitalized in “Sf. Ap. Andrei” St. Apostle Andrew’s County Emergency Hospital in various wards between March 2020 and August 2022. Data were collected from medical records and included inflammatory markers (C-reactive protein, procalcitonin, ESR) and coagulation markers (prothrombin time, INR, fibrinogen, D-dimer). The SPSS 2.0 statistical software was used to conduct the study. **Results:** *Coagulation markers*: Prothrombin activity averaged 74.22%, below normal levels, indicating a heightened bleeding risk, while fibrinogen levels were significantly elevated (mean: 531.69 mg/dL), reflecting hypercoagulability. Prolonged prothrombin time (mean: 17.28 s) and elevated INR (*International normalized ratio*) (mean: 1.51) were associated with increased mortality, emphasizing their role in risk stratification. Elevated D-dimer levels (mean: 2.75 mg/L) further highlighted thromboembolic risks. *Inflammatory markers*: C-reactive protein (CRP) and erythrocyte sedimentation rate (ESR) showed marked elevations (mean CRP: 92.09 mg/L, mean ESR: 58.47 mm/h), correlating with heightened systemic inflammation and poor outcomes. *Bacterial infections*: Elevated procalcitonin (mean: 1.98 ng/mL) suggested secondary bacterial infections, particularly in mechanically ventilated patients, significantly worsening prognosis. **Conclusions:** The duality of hypercoagulability and coagulopathy in elderly COVID-19 patients underscores the importance of consistently monitoring coagulation markers such as prothrombin time, INR, D-dimer, and fibrinogen. Simultaneously, elevated inflammatory markers and secondary bacterial infections require prompt therapeutic interventions. This study highlights the critical need for personalized management strategies to mitigate complications and reduce mortality in this high-risk population.

## 1. Introduction

This study is the result of the physician’s inherent curiosity, but above all, it stems from the love for humanity and life, values that define this noble profession. When we face illness and death, we do so with humility and decency, but never with indifference. A physician is someone who not only strives to do no harm but constantly seeks to find better ways to help.

Out of passion and empathy, we joined forces to confront an enemy that, perhaps for the first time, made us feel so close to one another. A virus made us more human by taking something from each of us: our peace of mind, our trust, or someone dear to us.

Driven by the fear of not having fully learned the lesson, we continue to research, to remain vigilant, but most importantly, to learn from our mistakes, if any were made.

The COVID-19 pandemic has had a significant impact on public health, particularly affecting elderly patients with comorbidities. In this context, it is paramount to understand the biological and immunological features influencing the course of the disease. Among these, inflammatory and coagulation factors play a central role in assessing the severity of infection and prognosis of patients. This article aims to provide a detailed retrospective review of laboratory results obtained from elderly patients hospitalized with COVID-19, analyzing parameters that may predict clinical course, including the risk of mortality and systemic decompensation.

The research hypothesis was chosen to clearly reflect the main objective of the study, emphasizing the importance of assessing biological parameters in the management and prognosis of elderly patients affected by COVID-19.

COVID-19 particularly affected this group of patients, known for their increased vulnerability to severe complications and high mortality. By including the terms “inflammatory” and “coagulation” in the research, the aim is to highlight those key parameters that provide valuable clues to disease progression and play a central role in monitoring the patients’ health status. These markers are frequently used to determine infection severity and to assess the systemic decompensation risk.

Also, using the word ’retrospective’ suggests a detailed analysis of existing data from laboratory results, providing an overview of how these parameters have influenced the clinical course. The research is intended to inform about the comprehensive nature of the study, emphasizing the complexity and depth of the analysis performed to provide readers with a full understanding of the relevant biomarkers in the context of this pathology.

## 2. Materials and Methods

The retrospective study was conducted on a sample of 1429 elderly patients (≥60 years) diagnosed with COVID-19, hospitalized in “Sf. Ap. Andrei” St. Apostle Andrew’s County Emergency Hospital in Romania, Galați city, various wards between March 2020 and August 2022. Data were collected from medical records and included inflammatory markers (C-reactive protein, procalcitonin, ESR) and coagulation markers (prothrombin time, INR, fibrinogen, D-dimer). The SPSS 2.0 statistical software was used to conduct the study.

Unfortunately, it was not possible to analyze the blood antithrombin levels, as this test is not routinely available in the laboratory of our healthcare facility due to the lack of reagents. The aPTT results were not included in the research because, at that time, this test was not performed for all patients. We mention that the lack of reagents for antithrombin and aPTT analysis was caused by the overwhelming number of cases of patients infected with COVID-19.

Regarding the method used, we mention that the immunoturbidimetric and coagulometric methods were employed.

Consequently, from a statistical perspective, we encountered a large amount of missing data, which would have led to incorrect interpretation of the final results. Regarding the prophylaxis used, enoxaparin sodium was administered. Regarding the treatment of elderly patients with COVID-19, it is worth mentioning that, in addition to the treatment of associated diseases, they received **prophylactic enoxaparin sodium** to reduce the risk of coagulopathy, and **dexamethasone** to decrease the risk of generalized inflammation by inhibiting the release of interleukin 1 and 2.

Additionally, **antiviral treatment with Remdesivir** (200 mg on day 1 and 100 mg for the second day) was administered. Patients who developed **bacterial pneumonia** simultaneously were treated with antibiotics according to the antibiogram, if their general condition allowed for a delay. Otherwise, they were treated empirically with **azithromycin**, **ceftriaxone**, or **cefoperazone with sulbactam**, depending on the clinician’s suspicion regarding the responsible pathogen.

Discharge status (death/survivor) was used as a dependent variable for statistically analyzing associations with biological parameters. The analysis included descriptive methods and statistical correlations to identify predictors of disease progression.

Normal values of the inflammatory and coagulation markers used in the present study:
Prothrombin time11–15 sInternational normalized ratio0.8–1.2D-dimer≤0.5 mg/LProthrombin activity70–130%Fibrinogen 1.5–4.5 g/LC-reactive protein<5 mg/LErythrocyte sedimentation rateMale: 0–15 mm/h; Female: 0–20 mm/hProcalcitonin<0.15 ng/mL


## 3. Results

### 3.1. Coagulation Markers

#### 3.1.1. Prothrombin Activity (%)

Prothrombin activity (%) is a measure of blood’s clotting ability, which is used to assess the function of the clotting system, particularly the liver, since prothrombin is a protein produced in the liver. This measurement is closely related to prothrombin time (PT), which measures how quickly a blood clot forms. Prothrombin activity (Table 1) expressed as a percentage reflects the comparison with a normal clotting level.

The interpretation of the descriptive statistics for prothrombin activity (75–110%) shows significant variability in the measured values, with a total of 493 observations. The minimum and maximum values, ranging between 8.7% and 150.5%, suggest a wide range of coagulation activity among the analyzed patients, which may indicate a heterogeneous distribution of their health status. The mean of 74.22% indicates that the mean values of prothrombin activity were in the lower end of the normal range but not extremely low.

The standard deviation of 23.58% shows a relatively large dispersion of values from the mean, indicating significant differences in coagulation function between patients. The negative skewness (−0.365) signals that the distribution is slightly shifted towards higher values of prothrombin activity, with a slightly higher number of cases having values above the mean. However, this skewness is not extreme. The flattening of 0.348 indicates a distribution fairly close to normal, with no extremely high or very low values, suggesting a slight clustering of the data around the mean, but not very pronounced.

The mean of 74.22% suggests a decreasing clotting activity in many patients, which may indicate a higher risk of bleeding or slow clotting, characteristic in severe forms of COVID-19.

A low prothrombin activity may signal an increased risk of bleeding complications, a particularly important aspect to consider when managing patients with COVID-19. Treatment of these patients often included anticoagulants to prevent thrombus formation, which made close monitoring of prothrombin activity essential. The low value also suggests that these patients may have a slower clotting response in situations of trauma or internal bleeding.

In the elderly, already having a reduced capacity for tissue regeneration and an increased susceptibility to severe complications due to inflammation and compromised immune response, a decrease in prothrombin activity may significantly increase the risk of death. This observation emphasizes the need for rigorous therapeutic intervention and constant monitoring of coagulation parameters in the management of these patients.

To interpret the association between prothrombin activity (75–110% ordered in three categories) transformed into a nominal variable and the variable deceased (Yes/No) (*Deceased patient or not, considering the discharge status: improved, recovered, deceased*), we analyze the contingency table provided. Table 2 shows the distribution of patients according to these two variables.

Prothrombin activity below the limit: Patients with subthreshold prothrombin activity (less than 75%) were significantly more likely to die (37.5%), indicating an association between low prothrombin activity and increased risk of death. This may be explained by the fact that low prothrombin activity reflects coagulation disorders, which can lead to severe complications in patients with COVID-19.

Normal prothrombin activity: Among patients with normal prothrombin activity (75–110%), the percentage of deaths is lower (23.7%). This suggests that patients with normal values have a lower risk of death, but the risk is not entirely eliminated.

Prothrombin activity above the limit: Patients with prothrombin activity above the limit have a very low risk of death (only 5%). Although this group is very small, there seems to be a correlation between high prothrombin activity and a favorable prognosis.

The results (Table 3) suggest a clear association between prothrombin activity and the probability of death. As prothrombin activity falls below normal, the risk of death increases significantly. This may be an important indicator for clinicians in monitoring and managing patients with COVID-19, especially those with coagulation disorders.

The results for Phi and Cramer’s V indicate a weak association between prothrombin activity (categories: below borderline, normal, above borderline) and death (Yes/No). The Phi coefficient has a value of 0.185, suggesting a weak relationship between these variables. Also, Cramer’s V, which adjusts Phi for larger contingency tables, has the same value of 0.185, indicating the same strength of association.

The statistical significance of this association is significant, with a *p*-value of 0.000, meaning that there is a statistically significant association between prothrombin activity and death. Although the association is weak, the fact that it is statistically significant suggests that prothrombin activity is nonetheless genuinely related to the risk of death, even if its influence is not very strong. This indicates that prothrombin activity may be only one of many factors contributing to the outcome and prognosis of patients with COVID-19.

#### 3.1.2. Prothrombin Time

Prothrombin time (PT) is a laboratory test used to assess the ability of blood to form clots. The test measures the time it takes for blood plasma to clot after the addition of certain chemicals that activate the clotting process. It is mainly used to assess the function of extrinsic and common coagulation factors, especially factors II, V, VII and X, as well as fibrinogen, a liver-produced protein.

The prothrombin time values (Table 4) were measured for a total of 493 patients and ranged from a minimum of 9.1 s to a maximum of 100.7 s. The calculated mean is 17.28 s, indicating that the mean prothrombin time in this sample is prolonged compared to the normal reference range (11–13 s). This suggests that many patients have significant impairment in coagulation ability.

The standard deviation of 9.44 s indicates significant variability among patients, which may reflect different stages of disease or severity of coagulation impairment.

The skewness of 4.476 indicates a highly skewed positive distribution, suggesting the presence of extremely high prothrombin time values for a small proportion of patients. This is also confirmed by the extremely high kurtosis value of 25.528, which indicates a distribution with a very sharp peak and long tails, signalling the presence of extreme cases in the sample.

The prolonged prothrombin time, with a mean of 17.28 s, well above the normal range of 11–13 s, reflects a significant dysfunction of the coagulation mechanism in elderly COVID-19 patients. This prolonged clotting time indicates that most patients have a slower response to blood clot formation, making them more susceptible to spontaneous bleeding or bleeding during invasive medical procedures.

COVID-19, especially in severe forms, impairs the coagulation system through intense systemic inflammation and dysregulated immune responses, leading to ineffective clotting. In the elderly, this problem is often aggravated by comorbidities such as high blood pressure, heart disease and diabetes, which can further impair the normal functioning of the coagulation process.

Prolonged prothrombin time also suggests that these patients may be at increased risk of developing complications such as sepsis-associated coagulopathy or acute respiratory distress syndrome (ARDS), two of the major complications seen in COVID-19. Careful monitoring of the prothrombin time becomes essential for proper adjustment of anticoagulant therapy and prevention of severe complications, including thromboembolism or massive bleeding.

In elderly patients with COVID-19, a prolonged prothrombin time may reflect not only the severity of the disease (Table 5), but also the need for a more aggressive therapeutic approach to prevent rapid progression to fatal complications.

The distribution of prothrombin time by death status shows a clear association between prolonged prothrombin time and risk of death. Patients with values below the normal limit of prothrombin time (less than 11 s) did not have any deaths, suggesting that they had a good clotting ability. In the group with normal values (11–13 s), there were 15 deaths out of a total of 106 patients, indicating a moderate risk of death.

Most patients were in the category with prothrombin time above the limit (over 13 s), where 250 survived but 130 died. This category suggests a strong association between prolonged prothrombin time and the probability of death. In conclusion (Table 6), patients with prolonged prothrombin time have a significantly higher risk of death, which emphasizes the importance of monitoring this parameter in assessing the severity of COVID-19.

The Phi coefficient value of 0.196 indicates a weak association between prothrombin time and death, suggesting a connection between the prolongation of prothrombin time and the probability of death, but the strength of this connection is not very strong. Cramer’s V has the same value, as the contingency table is almost symmetric, confirming an association of similar strength.

The statistical significance (*p* = 0.000) shows that this association is statistically significant, indicating that there is a real relationship between these variables and that it is not the result of chance. In conclusion, although the association is weak, the fact that it is statistically significant suggests that prothrombin time has some influence on the risk of death among the patients analyzed.

#### 3.1.3. INR (International Normalized Ratio)

INR (International Normalized Ratio) is an essential parameter used to assess blood coagulation function, particularly in the context of monitoring anticoagulant therapy and identifying coagulation dysfunction. In elderly patients with COVID-19, INR monitoring becomes extremely important, as they are at increased risk of thromboembolic complications and coagulopathies in the context of the severe inflammation and systemic damage that SARS-CoV-2 infection causes.

For healthy individuals, the INR is generally in the range of 0.8–1.2. An INR higher than this range suggests slower clotting (Table 7), which may increase the risk of bleeding, while a lower INR indicates faster clotting, associated with an increased risk of thrombosis. INR is essential for adjusting doses of anticoagulant drugs and preventing complications such as blood clots or bleeding.

INR values obtained from this sample of 493 patients show considerable variability, with a minimum of 0.73 and a maximum of 11.22. The mean INR is 1.5098, suggesting that, on average, patients have values higher than the normal range of 0.8–1.2, indicating slower-than-normal clotting for most patients, which may increase the risk of bleeding.

The standard deviation of 1.03 reflects a significant scatter around the mean, suggesting large differences in clotting ability between patients. The distribution shows a pronounced skewness, with a value of 4.981, indicating that there are a few very high INR values, pulling the distribution to the right. The flattening of 31,715 suggests a highly leptokurtic distribution, which means that the values are highly concentrated around the mean, but there are a few cases with values much higher than the mean.

The contingency table between INR (0.8–1.2, ordered in three categories) and deceased (Yes/No) (Table 8) shows the distribution of patients according to these two parameters. The results show a relationship between INR values and patient mortality.

Among patients with INR values below the normal limit (below 0.8), all four patients survived, suggesting that they were not at risk of death. In patients with normal INR values (0.8–1.2), 174 survived and 49 died, indicating a moderate risk of death, but lower than in the high-INR group. Most deaths occurred among patients with INR above the normal limit (above 1.2), where 96 out of 266 patients died. This suggests a significant association between elevated INR values and an increased risk of death.

These data indicate that patients with elevated INR are more prone to mortality, possibly due to inadequate coagulation or severe complications associated with coagulation system dysfunction, as frequently observed in COVID-19. Patients with normal or below-normal INR seem to have a more favorable prognosis compared to those with INR above the normal limit (Table 9).

The results of Phi and Cramer’s V coefficients indicate a weak association between INR values and death status. The Phi coefficient has a value of 0.164, which indicates that there is a relationship between these two variables, but the strength of this relationship is weak. Although the association is weak, the statistically significant value (*p* = 0.001) suggests that this relationship is real and not coincidental.

This indicates that despite a certain influence of INR values on the risk of death, it is not a very strong one. However, the fact that the association is significant indicates that the INR may have a relevant role in the outcome of patients, particularly those with COVID-19, but other variables are likely to have a significant contribution to this risk.

#### 3.1.4. D-Dimer

The D-Dimer test (*normal values* ≤ 0.5 mg/L) is commonly used to detect or monitor conditions that involve abnormal blood clot formation, such as deep vein thrombosis (DVT), pulmonary embolism or disseminated intravascular coagulation (DIC). In these conditions, D-Dimer levels are elevated due to the breakdown of fibrin that forms excess clots.

Descriptive results for D-Dimer (Table 10) show that, in the sample of 237 patients, values ranged from a minimum of ≤0.5 mg/L to a maximum of 12.53 mg/L, with a mean of 2.748 mg/L. This mean is well above the normal range (≤0.5 mg/L), indicating increased blood clot formation and degradation activity in most patients.

The standard deviation of 2.896 suggests significant variability in the D-Dimer values, reflecting large differences between patients in the risk of thrombosis or other coagulation complications. The positive skewness of 1.341 indicates a slightly skewed distribution, meaning that most values are concentrated at lower levels, but there are a significant number of cases with high values, which pull the mean upwards. The flattening of 0.889 indicates that the distribution is flatter than a normal distribution, suggesting the presence of several extreme values.

Overall, these data reflect an increased risk of thrombotic complications in patients, especially given the high and variable D-Dimer values, which may be relevant in the context of severe conditions such as COVID-19.

The contingency table between D-Dimer (normal and above limit) and death status (Table 11) shows a clear distribution of risk according to D-Dimer level. Of the patients with normal D-Dimer values (≤0.5 mg/L), 28 survived and 18 died, suggesting a relatively low risk of mortality in this group. In contrast, of patients with values above the normal limit, 120 survived but 71 died, indicating a strong association between increased D-Dimer elevation and an increased risk of death.

This difference highlights that patients with increased D-Dimer are significantly more likely to die than those with normal values. The presence of a D-Dimer above the normal limit reflects intense clotting and fibrinolytic activity, which is common in severe cases of COVID-19 or other serious conditions involving coagulation dysfunction. In conclusion, elevated D-Dimer levels are an important indicator of a poor prognosis, suggesting a direct connection between the values of this marker and patient mortality.

More specifically, by comparing the 18 deaths recorded among patients with normal D-Dimer values to the 71 deaths recorded among patients with D-Dimer levels above the normal limit, we can conclude that the risk of death increases with rising D-Dimer levels. Among patients with normal D-Dimer values, nearly 40% died, while in the group with elevated levels, mortality was similar, but the total number of severe cases was significantly higher.

The results for the Phi and Cramer’s V coefficients (Table 12) indicate a very weak association between D-Dimer values and death status, as the values of these coefficients are extremely small (Phi = −0.016 and Cramer’s V = 0.016). These values basically suggest a lack of significant association between the two variables. Also, the *p*-value of 0.806 indicates that this association is not statistically significant, which means that we cannot conclude that the D-Dimer level has a relevant impact on the probability of death in this dataset.

These results may indicate that, in this sample of elderly COVID-19 patients, D-Dimer levels do not have a strong or clear association with the risk of death or that other variables play a more important role in determining clinical outcomes.

#### 3.1.5. Fibrinogen

Fibrinogen is a protein produced by the liver and is essential in the blood clotting process. When an injury occurs in blood vessels, fibrinogen is converted into fibrin, which forms a network of strands that help stabilize blood clots. Fibrinogen thus plays a vital role in stopping bleeding and repairing tissues.

Normal fibrinogen in the blood is about 200–400 mg/dL, and deviations from this range may indicate medical problems. Elevated fibrinogen levels are associated with inflammatory conditions, infections, trauma or chronic diseases such as diabetes and cardiovascular disease. On the other hand, low levels may signal severe liver disease or clotting problems such as disseminated intravascular coagulation (DIC).

Descriptive data for fibrinogen in a sample of 700 patients (Table 13) show considerable variability. Fibrinogen values ranged from a low of 87.2 mg/dL to a high of 1477 mg/dL, indicating a wide range of fibrinogen levels among the patients analyzed. The mean fibrinogen is 531.69 mg/dL, which is above the normal reference range (200–400 mg/dL), suggesting that many patients have elevated fibrinogen levels, which may reflect inflammatory states or other coagulation-related conditions.

The standard deviation of 211.65 indicates a wide dispersion of fibrinogen values around the mean, signalling significant differences between patients. The positive skewness of 0.77 suggests that there are more values higher than the mean, pulling the distribution to the right. The kurtosis of 0.704 indicates that the distribution is relatively flat, with the presence of some extreme values, but without a pronounced concentration around the mean.

This distribution reflects the fact that many elderly patients with COVID-19 have elevated fibrinogen levels, a common indicator of inflammation and potential risk of thromboembolic complications.

The contingency table between fibrinogen levels (below limit, normal and above limit categories) and death status (Table 14) shows a relationship between fibrinogen levels and mortality risk. Of the patients with below-normal fibrinogen levels, a relatively small number were observed (thirteen survivors and nine deaths), which may suggest that these patients have impaired coagulation, but do not represent a significant group.

Among patients with normal fibrinogen levels, 132 survived, while 42 died. This group indicates a moderate risk of mortality compared to patients with elevated fibrinogen values.

The category of patients with fibrinogen above the normal limit is the largest and includes 504 patients, of whom 332 survived and 172 died. This suggests that patients with elevated fibrinogen have a higher risk of death, which may reflect the presence of severe systemic inflammation or coagulation-related complications, which are common in severe cases of COVID-19.

This distribution suggests an association between elevated fibrinogen levels and mortality, with patients with values above the normal limit having a significantly higher risk of death compared to those with normal or below-normal fibrinogen.

The results of Phi and Cramer’s V coefficients (Table 15) show a weak association between fibrinogen levels and death status, with a value of 0.099 for both measures. This association is statistically significant, with a *p*-value of 0.033, indicating that there is a real relationship between the variables and that it is not random. Although the association is significant, its strength is poor, suggesting that although fibrinogen may influence the risk of death in elderly patients with COVID-19 to some extent, it is not a strong determinant, and other factors are likely to have a significant contribution to mortality.

### 3.2. Inflammatory Markers

#### 3.2.1. Procalcitonin

Procalcitonin (PCT) is a protein biomarker produced by the body’s cells in response to severe bacterial infection or systemic inflammation. Under normal conditions, procalcitonin levels are very low or almost undetectable in the blood. In the presence of a bacterial infection, procalcitonin is released into the bloodstream, where it can be measured to assess the severity of the infection. The rapid rise in procalcitonin is triggered in particular by systemic bacterial infections, such as sepsis, and is considered an early and sensitive indicator of these conditions (Tocu et al., 2023) [1].

The procalcitonin test is mainly used to help differentiate between bacterial and viral infections since viral infections do not lead to significant increases in this biomarker. The test can also be used to assess the need for antibiotic treatment, to monitor the progress of a severe infection or to determine the risk of complications such as sepsis.

Elevated procalcitonin values indicate the presence of a severe bacterial infection or a severe inflammatory response, while low or normal levels suggest a viral infection or the absence of a significant bacterial infection. This test is particularly useful in the context of respiratory infections or critically ill patients who are susceptible to sepsis or other systemic bacterial infections.

Descriptive data for procalcitonin (Table 16) in a sample of 269 patients indicate significant variability in the levels of this biomarker. Procalcitonin values ranged from a low of 0.03 ng/mL to a high of 43.9 ng/mL, reflecting large differences in the severity of inflammation or bacterial infection among patients. The mean of 1.98 ng/mL is well above the normal reference range (0–0.1 ng/mL), suggesting that many patients have elevated procalcitonin levels, which may indicate the presence of severe bacterial infections or systemic inflammation.

The standard deviation of 6.15 shows a wide dispersion of values, confirming the presence of large differences between patients. The positive skewness of 4.54 indicates a strongly skewed distribution, with a relatively small number of patients having extremely high levels of procalcitonin, which pulls the mean upwards. The flattening of 21.102 indicates a leptokurtic distribution, with a high concentration of values around the mean, but also with the presence of extreme values.

These data suggest that the majority of patients in this sample have procalcitonin levels above the normal limit, which may reflect the presence of severe bacterial infections or significant inflammation.

The contingency (Table 17) between procalcitonin level (normal and above limit) and death status shows a significant difference between the two groups. Of the patients with normal procalcitonin levels (0–0.1 ng/mL), 72 survived and 31 died. In contrast, among those with levels above the normal limit, 92 survived and 74 died.

This suggests that patients with elevated procalcitonin levels have a higher risk of death compared to those with normal levels. High levels of procalcitonin are usually associated with severe bacterial infections and systemic inflammation, factors that can worsen patients’ condition and increase the risk of mortality.

The results of the Phi and Cramer’s V coefficients (Table 18) show a weak association between procalcitonin levels and death status, with a value of 0.144 for both measures. Although the association is weak, statistical significance (*p* = 0.018) indicates that this association is statistically significant and not the result of chance. This suggests that although increased levels of procalcitonin are associated with a higher risk of death, the relationship is not a very strong one. However, procalcitonin may be an important indicator of disease severity, particularly in elderly patients with COVID-19.

#### 3.2.2. C-Reactive Protein

C-reactive protein (CRP) is a protein produced by the liver that appears in the blood in response to inflammation. The C-reactive protein test measures the levels of this protein to detect inflammation in the body. CRP levels are normally low but increase significantly in the presence of acute or chronic inflammation, infection, trauma or certain serious diseases. CRP is a non-specific marker that indicates the presence of inflammation but does not give precise details about its cause.

Elevated CRP levels are found in many conditions, from bacterial or viral infections to chronic inflammatory diseases and cardiovascular diseases. In the context of COVID-19 infection, CRP is an important indicator of disease severity, reflecting strong systemic inflammation. Monitoring CRP levels in these patients can help assess disease severity and guide treatment.

Descriptive data for C-reactive protein (CRP) (Table 19) in a sample of 780 patients show significant variability, with values ranging from a low of 0.6 mg/L to a high of 450.5 mg/L. The mean of 92.09 mg/L is well above the normal range (0–10 mg/L), indicating that most patients have very high CRP levels, suggesting the presence of severe inflammation or acute infection.

The 79.49 standard deviation shows a large scatter around the mean, reflecting large differences in inflammatory response between patients. The positive skewness of 1.044 indicates a slightly skewed distribution, with higher CRP values recorded for a small number of patients, which pulls the distribution to the right. The kurtosis of 1.104 indicates a leptokurtic distribution, suggesting a concentration of values around the mean, but also the presence of extremely high values.

These results suggest that patients in this sample have severe inflammation, reflected by the elevated CRP values, which may be associated with severe infections, such as COVID-19, or other conditions that trigger strong inflammatory responses.

The contingency table for C-reactive protein and death status (Table 20) shows a clear relationship between C-reactive protein levels and mortality. Of the patients with normal C-reactive protein levels (0–10 mg/L), 102 survived, while only 16 died. In contrast, in the group of patients with levels above the normal limit, 408 survived and 254 died.

These data suggest that patients with elevated levels of C-reactive protein have a significantly higher risk of death than those with normal values. Elevated levels of C-reactive protein indicate severe systemic inflammation, which may worsen the patients’ condition and contribute to a poor prognosis, particularly in the context of serious conditions such as COVID-19.

The results of Phi and Cramer’s V coefficients in Table 21 indicate a small association between C-reactive protein levels and death status. A Phi and Cramer’s V value of 0.187 suggests a significant association between these two variables, and a *p*-value of 0.000 indicates that this association is highly statistically significant. This indicates that elevated C-reactive protein levels are associated with an increased risk of death, confirming that severe systemic inflammation, as reflected by elevated C-reactive protein values, may contribute to a poorer prognosis among elderly patients with COVID-19.

#### 3.2.3. ESR (Erythrocyte Sedimentation Rate)

ESR (erythrocyte sedimentation rate) is a laboratory test that measures how quickly red blood cells (erythrocytes) settle in a blood sample. It is a non-specific indicator of inflammation in the body. Under normal conditions, red blood cells settle slowly to the bottom of the test tube. When there is inflammation, certain proteins in the blood (such as fibrinogen) cause the red blood cells to clump together and settle more quickly. Thus, an elevated ESR suggests the presence of inflammation or infection but does not indicate the precise source.

ESR is commonly used to help diagnose or monitor chronic inflammatory conditions such as rheumatoid arthritis, lupus, infections and some cancers. However, because it is a non-specific marker, it is often used in conjunction with other laboratory tests to provide a complete diagnosis.

ESR (erythrocyte sedimentation rate) is a nonspecific marker of inflammation, but it is also closely linked to the coagulation process, as fibrinogen (a coagulation protein) plays a central role in erythrocyte aggregation. Elevated fibrinogen levels, observed in acute inflammation or chronic diseases, lead to accelerated sedimentation of red blood cells, which explains the high ESR values under these conditions.

Thus, ESR may indicate increased erythrocyte aggregation in the presence of acute-phase proteins, risk of microthrombosis due to fibrinogen involvement in blood cell aggregation, chronic inflammatory states or autoimmune diseases, where ESR is often elevated.

Descriptive data for ESR (erythrocyte sedimentation rate) (Table 22) in a sample of 1058 patients show significant variability, with values ranging from a minimum of 5 mm/h to a maximum of 141 mm/h. The mean of 58.47 mm/h is well above the normal reference range (2–15 mm/h), suggesting that most patients have an elevated ESR, indicating inflammation or infection in the body.

The standard deviation of 36.41 shows a considerable scatter around the mean, reflecting large variations between patients. The positive skewness of 0.386 indicates a slight skewness of the distribution, with several large values pulling the distribution to the right. Negative kurtosis (−0.885) suggests a relatively flat distribution with fewer pronounced peaks, indicating the presence of extreme values, but more evenly distributed.

These results suggest that a large number of patients have systemic inflammation or infection, which may reflect the severity of the disease, particularly in the context of elderly patients with COVID-19, where inflammation is a major risk factor.

The contingency table for ESR (erythrocyte sedimentation rate) and death status (Table 23) indicates a significant difference between patients with normal levels and those with values above the normal limit. Of the patients with normal ESR (2–15 mm/h), 126 survived and 33 died. In contrast, among patients with ESR above the normal limit, 603 survived and 296 died.

This distribution shows that patients with an elevated ESR have a much higher risk of death than those with normal values. An elevated ESR usually reflects significant inflammation in the body, which is frequently associated with severe conditions, including systemic infections and serious illness. These data suggest that patients with elevated ESR have a poorer outcome with an increased risk of mortality, which is particularly relevant in the context of elderly patients with COVID-19.

The results of the Phi and Cramer’s V coefficients in Table 24 indicate a weak association between ESR values and death status, with a value of 0.094 for both measures. Although the strength of the association is low, the statistical significance (*p* = 0.002) indicates that the relationship between ESR and mortality is statistically significant, which means that there is a real relationship between these two variables.

This suggests that patients with elevated ESR values have a slightly increased risk of death, but ESR alone is not a strong predictor of mortality. Other variables or additional factors probably play a more important role in determining patients’ prognosis. However, ESR may provide an additional indication of the severity of the inflammatory state, especially in elderly patients with COVID-19.

## 4. Discussions

The results obtained in this study emphasize the importance of rigorous evaluation of coagulation and inflammatory markers in elderly patients with COVID-19, as they play a crucial role in determining clinical course and prognosis. Our findings align with the recent literature emphasizing the frequency and severity of coagulopathy and systemic inflammation in COVID-19, contributing to a thorough understanding of these pathophysiological processes in the elderly population, one of the most vulnerable to severe forms of the disease.

Guzman N et al. (2024)—J Clin Med [2] develop a predictive model for mortality in patients hospitalized with COVID-19 by analyzing various clinical and laboratory parameters to estimate the risk of death during hospitalization. Our study aligns with the results of the mentioned study and aims to contribute to this field, particularly considering that the statistical program used forms the basis for artificial intelligence algorithms.

The study by Jensen TO et al. (2024)—Lancet [3] Microbe track the evolution of virological and immunological biomarkers in patients hospitalized with COVID-19 [4], identifying associations between these biomarkers and clinical outcomes, including mortality and severe complications. The findings are similar to those in our study regarding COVID-19-associated coagulopathies.

Similarly to the results obtained in this study, Violi F et al. (2021)—Intern Emerg Med [4] investigate the relationship between arterial and venous thrombosis and mortality in patients with COVID-19, highlighting that thrombosis is a major factor contributing to an increased risk of death.

The study by Iqbal Q et al. (2024)—Narra J [5] analyzes hemostatic parameters and liver function as markers of COVID-19 severity, showing that abnormalities in these parameters are correlated with an increased risk of complications and mortality.

The study by Koutsiaris et al., Clinical Hemorheology and Microcirculation, 2022 [6] investigated capillary thrombosis in the bulbar conjunctiva using high-resolution imaging to evaluate ocular microcirculation. The authors quantified blood flow and shear stress at the vascular wall level in microvessels with diameters ranging from 4 to 24 micrometers. The results showed significant variations in these parameters, suggesting the presence of microthrombosis and impaired local hemodynamics. These findings emphasize the importance of ocular microcirculation monitoring in assessing coagulation status in patients with COVID-19.

The study by Rosei et al., Journal of Hypertension, 2022 [7] focused on evaluating capillary thrombosis in the nailfold area using periungual capillaroscopy to detect microvascular abnormalities. The authors observed structural and functional changes in the capillaries, including dilations, microcapillary hemorrhages, and reduced capillary density, indicating the presence of microthrombosis. These changes were correlated with the severity of COVID-19 infection, suggesting that capillaroscopic evaluation could serve as a diagnostic and prognostic tool in managing affected patients.

The study by Koutsiaris, Life, 2024 [8] proposes a blood flow reduction mechanism in COVID-19, attributing microvascular loss to extensive microthrombosis. The mathematical models and clinical observations presented suggest that microthrombosis leads to a significant decrease in tissue perfusion, contributing to hypoxia and organ damage. This mechanism partially explains why some patients develop severe complications and highlights the need for therapeutic interventions targeting the prevention and treatment of microthrombosis in COVID-19.

These studies highlight the crucial role of microthrombosis in the pathogenesis of COVID-19 and the importance of non-invasive imaging methods, such as capillaroscopy, for early detection and monitoring of these microvascular changes.

In our analysis, the prothrombin activity showed a mean of 74.22%, at the lower limit of the normal range, indicating a significant impairment of coagulation capacity. Patients with values below 75% had a mortality rate of 37.5% compared to only 5% for those with values above 110%. This phenomenon is supported by numerous studies highlighting that coagulation deficiencies, reflected by reduced prothrombin activity, are associated with an increased risk of fatal complications in patients with COVID-19 (Connors & Levy, 2020) [9]. This association is of particular importance in elderly patients, where impaired coagulation is frequently exacerbated by pre-existing comorbidities such as hypertension and cardiovascular disease.

Another relevant marker is the prothrombin time, which averaged 17.28 s, significantly prolonged from the normal range (11–13 s). This prolonged clotting time was associated with a much higher mortality risk, suggesting a severe impairment of coagulation mechanisms. Indeed, the literature emphasizes that patients with COVID-19, especially those with severe forms, frequently present with disseminated intravascular coagulopathy (DIC), characterized by prolonged prothrombin time and a high risk of bleeding and thrombosis (Tang et al., 2020) [10]. These coagulation dysfunctions are central to understanding morbidity and mortality in elderly patients with COVID-19.

The INR (International Normalized Ratio) averaged 1.5098, reflecting abnormally slow coagulation. Although the association between INR and mortality was relatively weak (Phi = 0.164), it was statistically significant (*p* = 0.001), emphasizing that elevated INR may be an important indicator of worsening clinical conditions. Previous studies have shown that increased INR is frequently associated with an increased risk of bleeding and thrombotic complications in critically ill patients, including those with COVID-19 (Barrett et al., 2020) [11].

D-Dimer, an important marker of fibrinolytic activity and blood clot formation, showed an elevated mean of 2.748 mg/L, more than five times higher than the normal maximum value (0.5 mg/L). These elevated levels are consistent with the literature suggesting that elevated D-Dimer is an important predictor of thrombosis and severe complications in patients with COVID-19, particularly among those with severe forms of the disease (Helms et al., 2020) [12]. Although, in this study, the association between D-Dimer and mortality was not statistically significant (*p* = 0.806), we consider that these statistical results may be attributed to the fact that the studied groups, namely recovered patients versus deceased patients, were not equally sized, suggesting also that other factors may contribute more strongly to the risk of death in this setting (comorbidities, lifestyle, genetic and psychological factors, the patient’s promptness in seeking medical care, and the anticoagulant treatment administered).

Fibrinogen, with a mean of 531.69 mg/dL, reflects the severe systemic inflammation characteristic of patients with severe forms of COVID-19. Fibrinogen is known to play an essential role in the coagulation process and inflammatory response, and elevated levels have been associated with an increased risk of thrombosis and mortality (Zhang et al., 2020) [13]. Consistent with these data, in our study, patients with elevated fibrinogen had significantly higher mortality (*p* = 0.033), which emphasizes the importance of monitoring this marker in elderly patients with COVID-19.

Regarding inflammatory markers, procalcitonin was an important indicator of disease severity, with mean values well above the normal limit (1.98 ng/mL versus < 0.1 ng/mL). These patients had an increased mortality rate, and procalcitonin was significantly associated with the risk of death (*p* = 0.018). This observation is in agreement with studies suggesting that procalcitonin is a key marker of secondary bacterial infections, which aggravate systemic inflammation in patients with COVID-19 (Hu et al., 2020) [14].

C-reactive protein (CRP) was also a key inflammatory marker, with a mean of 92.09 mg/L, well above the normal value (0–10 mg/L). Elevated CRP levels correlated with severe systemic inflammation and had a statistically significant association with mortality (*p* = 0.000). CRP is well known for its role in the prognosis of patients with COVID-19 and is a recognized predictor of severe disease progression (Shao et al., 2020) [15].

ESR (erythrocyte sedimentation rate), with a mean of 58.47 mm/h, reflected extensive systemic inflammation associated with an increased risk of death. Although the association was relatively weak (Phi = 0.094), the statistical significance (*p* = 0.002) emphasizes the role of inflammation in determining the prognosis of elderly patients with COVID-19 (Guan et al., 2020) [16].

## 5. Conclusions

In conclusion, these results emphasize that coagulation and inflammatory markers are essential predictors of the prognosis of elderly patients with COVID-19. Elevated levels of these markers were correlated with an increased risk of mortality and severe complications, confirming the need for close monitoring and early therapeutic interventions. These findings emphasize the importance of using these biomarkers to guide treatment strategies and prevent severe outcomes in vulnerable patients.

In elderly patients infected with COVID-19, the clinical course is often marked by a complex interaction between hypercoagulation and coagulopathy, which are essential factors in determining the risk of severe complications and mortality. Analysis of coagulation markers has demonstrated significant variations, highlighting the importance of constant assessment of prothrombin activity, prothrombin time, INR, D-Dimer and fibrinogen for the rapid identification of patients at risk.

**The contradiction between hypercoagulation and coagulopathy**: While prothrombin activity averaged 74.22%, below normal, indicating reduced coagulation and increased risk of bleeding, some patients had significantly elevated fibrinogen values (mean 531.69 mg/dL), suggesting a hypercoagulable state. This duality reflects a paradoxical phenomenon of coagulation in COVID-19, in which patients may be both at risk of bleeding due to coagulopathy and at risk of thrombosis due to hypercoagulation.

**Prothrombin time and INR**: Prolonged values of prothrombin time (mean 17.28 s) and INR above normal (mean 1.51) indicated slowed coagulation in many patients, which was associated with increased mortality. The high standard deviation and high skewness suggested the presence of patients with severe coagulation disorders, and the statistical associations between these markers and the risk of death were significant. Patients with prothrombin time over 13 s and INR over 1.2 were significantly more likely to develop fatal complications, reflecting the importance of these markers in risk stratification.

**D-Dimer and fibrinogen**: Elevated levels of D-Dimer (mean 2.75 mg/L) and fibrinogen were correlated with an activation of the coagulation system, indicating an increased risk of thromboembolism in elderly patients. These increases reflect hypercoagulation and are commonly associated with an increased risk of deep vein thrombosis and pulmonary embolism. These patients had a high mortality, emphasizing the need for constant monitoring of these parameters during disease progression.

**Secondary bacterial infections**: The very high procalcitonin values (mean 1.98 ng/mL, well above the normal limit of 0.1 ng/mL) suggested the presence of secondary bacterial infections, which are common among COVID-19 patients requiring mechanical ventilation or other forms of intensive support. These infections significantly worsen the prognosis, and patients with elevated procalcitonin had significantly higher mortality. Similarly, bacterial infections evidenced by increased numbers of bacteria and bacilli in laboratory samples suggested a severe complication of the initial viral infection, often lethal in the absence of aggressive antibiotic treatment.

**Inflammatory markers**: C-reactive protein and ESR, markers of systemic inflammation, were present at extremely high levels in most cases (mean CRP 92.09 mg/L and mean ESR 58.47 mm/h), indicating an exaggerated inflammatory response associated with COVID-19. Elevated levels of these markers were directly correlated with increased mortality, reflecting the impact of cytokine storm syndrome on elderly patients. The uncontrolled inflammation contributed to the rapid deterioration in the health status of these patients and was an important indicator of the need for rapid therapeutic intervention, including aggressive anti-inflammatory treatments.

COVID-19 is known to cause both hypo-coagulation (bleeding tendency) and hyper-coagulation (tendency to form blood clots). This paradoxical duality may seem contradictory but represents an important feature of COVID-19-associated coagulopathy, which can have multiple explanations, including the administration of antithrombotic medication and the possibility of the existence of an unknown coagulation factor, as proposed by Koutsiaris et al. (2022) [6].

The contradiction between hypo-coagulation and hyper-coagulation in COVID-19 can be explained by the administration of antithrombotic medication, which reduces the risk of thrombosis but increases the risk of bleeding, excessive consumption of coagulation factors during the process of microthrombosis and the existence of an unknown coagulation factor, which could play a central role in the development of microthrombosis.

This paradoxical duality highlights the importance of constant monitoring of coagulation markers and the personalization of anticoagulant treatment according to each patient’s condition.

Our study clearly demonstrates that careful and constant monitoring of inflammatory and coagulation markers is essential for the effective management of elderly patients with COVID-19. Understanding the contradiction between hypercoagulation and coagulopathy, identifying secondary bacterial infections and monitoring inflammation are critical steps in reducing the risk of severe complications and mortality in this vulnerable population. These findings underscore the need for personalized therapeutic strategies tailored to each patient’s individual risk profile.

## Figures and Tables

**Table 1:** Descriptive statistics—prothrombin activity.

Descriptive Statistics	N	Min	Max	Mean	Std. Dev.	Skewness		Kurtosis	
						Stat.	Std. Error	Stat.	Std. Error
Prothrombin activity %	493	8.7	150.5	74.22	23.58	−0.365	0.11	0.348	0.22
Valid N (listwise)	493								

**Table 2:** Contingency table—prothrombin activity * deceased.

Prothrombin Activity 75–110% Ord * Deceased Cross-Tabulation		Deceased		Total
		No	Yes	
Prothrombin activity 75–110% Ord	Below the limit	145	87	232
	Normal	184	57	241
	Above the limit	19	1	20
Total		348	145	493

**Table 3:** Extent of association—prothrombin activity * deceased.

Symmetric Measures		Value	Approx. Sig.
Nominal by Nominal	Phi	0.185	0.000 ^a^
	Cramer’s V	0.185	0.000 ^a^
N of Valid Cases		493	

^a^ Not assuming the null hypothesis (approx. sig. < 0.05).

**Table 4:** Descriptive statistics—prothrombin time.

Descriptive Statistics	N	Min	Max	Mean	Std. Dev.	Skewness		Kurtosis	
						Stat.	Std. Error	Stat.	Std. Error
Prothrombin time 11–13 s	493	9.1	100.7	17.28	9.44	4.476	0.11	25.528	0.22
Valid N (listwise)	493								

**Table 5:** Contingency table—prothrombin time * deceased.

Prothrombin Time 11–13 s Ord * Deceased Crosstabulation		Deceased		Total
		No	Yes	
Prothrombin time 11–13s Ord	Below the limit	7	0	7
	Normal	91	15	106
	Above the limit	250	130	380
Total		348	145	493

**Table 6:** Extent of association—prothrombin time * deceased.

Symmetric Measures		Value	Approx. Sig.
Nominal by Nominal	Phi	0.196	0.000 ^a^
	Cramer’s V	0.196	0.000 ^a^
N of Valid Cases		493	

^a^ Not assuming the null hypothesis.

**Table 7:** Descriptive statistics—INR.

Descriptive Statistics	N	Min	Max	Mean	Std. Dev.	Skewness		Kurtosis	
						Stat.	Std. Error	Stat.	Std. Error
INR 0.8–1.2	493	0.73	11.22	1.5098	1.03	4.981	0.11	31.715	0.22
Valid N (listwise)	493								

**Table 8:** Contingency table—INR * deceased.

INR 0.8–1.2 Ord * Deceased Crosstabulation		Deceased		Total
		No	Yes	
INR 0.8–1.2 Ord	Below the limit	4	0	4
	Normal	174	49	223
	Above the limit	170	96	266
Total		348	145	493

**Table 9:** Extent of association—INR * deceased.

Symmetric Measures		Value	Approx. Sig.
Nominal by Nominal	Phi	0.164	0.001 ^a^
	Cramer’s V	0.164	0.001 ^a^
N of Valid Cases		493	

^a^ Not assuming the null hypothesis.

**Table 10:** Descriptive statistics—D-Dimer.

Descriptive Statistics	N	Min	Max	Mean	Std. Dev.	Skewness		Kurtosis	
						Stat.	Std. Error	Stat.	Std. Error
D-Dimer ≤ 0.5 mg/L	237	≤0.5	12.53	2.748	2.896	1.341	0.158	0.889	0.315
Valid N (listwise)	237								

**Table 11:** Contingency table—D-Dimer * deceased.

D-Dimer ≤ 0.5 mg/L Ord * Deceased Crosstabulation		Deceased		Total
		No	Yes	
D-Dimer ≤ 0.5 mg/L Ord	Normal	28	18	46
	Above the limit	120	71	191
Total		148	89	237

**Table 12:** Extent of association—D-Dimer * deceased.

Symmetric Measures		Value	Approx. Sig.
Nominal by Nominal	Phi	−0.016	0.806 ^b^
	Cramer’s V	0.016	0.806 ^b^
N of Valid Cases		237	

^b^ Using the asymptotic standard error assuming the null hypothesis.

**Table 13:** Descriptive statistics—fibrinogen.

Descriptive Statistics	N	Min	Max	Mean	Std. Dev.	Skewness		Kurtosis	
						Stat.	Std. Error	Stat.	Std. Error
Fibrinogen 200–400 mg/dL	700	87.2	1477	531.69	211.65	0.77	0.092	0.704	0.185
Valid N (listwise)	700								

**Table 14:** Contingency table—fibrinogen * deceased.

Fibrinogen 200–400 mg/dL Ord * Deceased Crosstabulation		Deceased		Total
		No	Yes	
Fibrinogen 200–400 mg/dL Ord	Below the limit	13	9	22
	Normal	132	42	174
	Above the limit	332	172	504
Total		477	223	700

**Table 15:** Extent of association—fibrinogen * deceased.

Symmetric Measures		Value	Approx. Sig.
Nominal by Nominal	Phi	0.099	0.033 ^a^
	Cramer’s V	0.099	0.033 ^a^
N of Valid Cases		700	

^a^ Not assuming the null hypothesis

**Table 16:** Descriptive statistics—procalcitonin.

Descriptive Statistics	N	Min	Max	Mean	Std. Dev.	Skewness		Kurtosis	
						Stat.	Std. Error	Stat.	Std. Error
Procalcitonin 0–0.1 ng/mL	269	0.03	43.9	1.98	6.15	4.54	0.149	21.102	0.296
Valid N (listwise)	269								

**Table 17:** Contingency table—procalcitonin * deceased.

Procalcitonin 0–0.1 ng/mL Ord * Deceased Crosstabulation		Deceased		Total
		No	Yes	
Procalcitonin 0–0.1 ng/mL Ord	Normal	72	31	103
	Above the limit	92	74	166
Total		164	105	269

**Table 18:** Extent of association—procalcitonin * deceased.

Symmetric Measures		Value	Approx. Sig.
Nominal by Nominal	Phi	0.144	0.018 ^a^
	Cramer’s V	0.144	0.018 ^a^
N of Valid Cases		269	

^a^ Not assuming the null hypothesis.

**Table 19:** Descriptive statistics—C-reactive protein.

Descriptive Statistics	N	Min	Max	Mean	Std. Dev.	Skewness		Kurtosis	
						Stat.	Std. Error	Stat.	Std. Error
C-reactive protein 0–10 mg/L	780	0.6	450.5	92.09	79.49	1.044	0.088	1.104	0.175
Valid N (listwise)	780								

**Table 20:** Contingency table—C-reactive protein * deceased.

C-Reactive Protein 0–10 mg/L Ord * Deceased Crosstabulation		Deceased		Total
		No	Yes	
C-reactive protein 0–10 mg/L Ord	Normal	102	16	118
	Above the limit	408	254	662
Total		510	270	780

**Table 21:** Extent of association—C-reactive protein * deceased.

Symmetric Measures		Value	Approx. Sig.
Nominal by Nominal	Phi	0.187	0.000 ^a^
	Cramer’s V	0.187	0.000 ^a^
N of Valid Cases		780	

^a^ Not assuming the null hypothesis.

**Table 22:** Descriptive statistics—ESR.

Descriptive Statistics	N	Min	Max	Mean	Std. Dev.	Skewness		Kurtosis	
						Stat.	Std. Error	Stat.	Std. Error
ESR 2–15 mm/h	1058	5	141	58.47	36.41	0.386	0.075	−0.885	0.15
Valid N (listwise)	1058								

**Table 23:** Contingency table—ESR 2–15 mm/h Ord * deceased.

ESR 2–15 mm/h Ord * Deceased Crosstabulation		Deceased		Total
		No	Yes	
ESR 2–15 mm/h Ord	Normal	126	33	159
	Above the limit	603	296	899
Total		729	329	1058

**Table 24:** Extent of association—ESR * deceased.

Symmetric Measures		Value	Approx. Sig.
Nominal by Nominal	Phi	0.094	0.002 ^a^
	Cramer’s V	0.094	0.002 ^a^
N of Valid Cases		1058	

^a^ Not assuming the null hypothesis.

## Data Availability

The data presented in this study are available on request from the corresponding author.
